# Development and Morphology of the Ventricular Outflow Tracts

**DOI:** 10.1177/2150135116651114

**Published:** 2016-09-01

**Authors:** Robert H. Anderson, Shumpei Mori, Diane E. Spicer, Nigel A. Brown, Timothy J. Mohun

**Affiliations:** 1 Institute of Genetic Medicine, Newcastle University, Newcastle upon Tyne, United Kingdom; 2 Division of Cardiovascular Medicine, Department of Internal Medicine, Kobe University Graduate School of Medicine, Kobe, Japan; 3 Department of Pediatric Cardiology, University of Florida, Gainesville, FL, USA; 4 Johns Hopkins All Children’s Heart Institute, St Petersburg, FL, USA; 5 Division of Biomedical Sciences, St George’s, University of London, London, United Kingdom; 6 Division of Developmental Biology, MRC National Institute for Medical Research, London, United Kingdom

**Keywords:** truncus, conus, conotruncal anomalies, normal anatomy, episcopic microscopy, multidetector computer tomography

## Abstract

It is customary, at the current time, to consider many, if not most, of the lesions involving the ventricular outflow tract in terms of conotruncal malformations. This reflects the introduction, in the early 1940s, of the terms conus and truncus to describe the components of the developing outflow tract. The definitive outflow tracts in the postnatal heart, however, possess three, rather than two, components. These are the intrapericardial arterial trunks, the arterial roots, and the subvalvar ventricular outflow tracts. Congenital lesions afflicting the arterial roots, however, are not currently considered to be conotruncal malformations. This suggests a lack of logic in the description of cardiac development and its use as a means of categorizing congenital malformations. It is our belief that the developing outflow tract, like the postnatal outflow tracts, can readily be described in tripartite fashion, with its distal, intermediate, and proximal components forming the primordiums of the postnatal parts. In this review, we present evidence obtained from developing mice and human hearts to substantiate this notion. We show that the outflow tract, initially with a common lumen, is divided into its aortic and pulmonary components by a combination of an aortopulmonary septum derived from the dorsal wall of the aortic sac and outflow tract cushions that spiral through its intermediate and proximal components. These embryonic septal structures, however, subsequently lose their septal functions as the outflow tracts develop their own discrete walls. We then compare the developmental findings with the anatomic arrangements seen postnatally in the normal human heart. We show how correlations with the embryologic findings permit logical analysis of the congenital lesions involving the outflow tracts.

## Introduction

In a seminal work published nearly three-quarters of a century ago, Kramer, when considering the extant knowledge regarding the development of the outflow tracts, pointed to the need for “a redefinition of the terms used in describing the structures involved.”^1^ This was, in part, because of the “discrepant terms employed by previous workers.” Equally important, in his opinion, was the influence of “the rapidly changing shapes and locations in which the structures themselves are found at different stages of development.” Kramer described important new findings in his own contribution, not least the appearance of the intercalated cushions. As he showed, it was these structures that formed the primordiums of the arterial valves. It is questionable, however, whether the terms he coined to solve the problem of previous discrepancies, namely, “truncus” and “conus,” achieved the understanding he was seeking. This is because, as far as we are aware, there is no current agreement as to whether the arterial roots, formed by incorporating his newly described intercalated cushions, belong to the truncus or the conus. His terms, nonetheless, have achieved widespread acceptance, leading to the ongoing description of “conotruncal anomalies.” This practice is not without its own problems, since lesions of the arterial valves, such as the bicuspid aortic valve, the commonest congenital cardiac malformation,^2^ are not currently classified within the “conotruncal” category. Other lesions, in contrast, such as discordant atrioventricular connections, are included by some as representing conotruncal malformations,^3^ even though the primary process underlying their formation involves abnormal ventricular looping. The major reason why the suggestions of Kramer, important as they have been, have not matched his expectations is that the postnatal outflow tracts have three, rather than two, components. These are the intrapericardial arterial trunks, the arterial valvar leaflets and their supporting sinuses, collectively forming the arterial roots, and the subvalvar ventricular outflow tracts. When assessed in such tripartite fashion, development can be described in a fashion that permits direct correlations to be made with the multiple congenital cardiac lesions known to afflict the outflow tracts.^4-6^ In this review, we show how the tripartite approach clarifies knowledge concerning both the development and morphology of the ventricular outflow tracts.

## Development of the Heart Tube

Our knowledge of cardiac development has changed immeasurably over the past two or three decades. In part, this has depended on the advances made by molecular biologists. These moves have been matched by an increased ability to examine the anatomy of the developing heart using techniques such as scanning electron microscopy or high-resolution episcopic microscopy.^7^ The latter opportunities have revolutionized our capabilities of visualizing the morphological changes. Use of these techniques confirms that the outflow tract develops in a tripartite fashion,^5,6^ permitting more obvious appreciation of the changes that occur early in the development of the heart tube.

**Figure 1.**
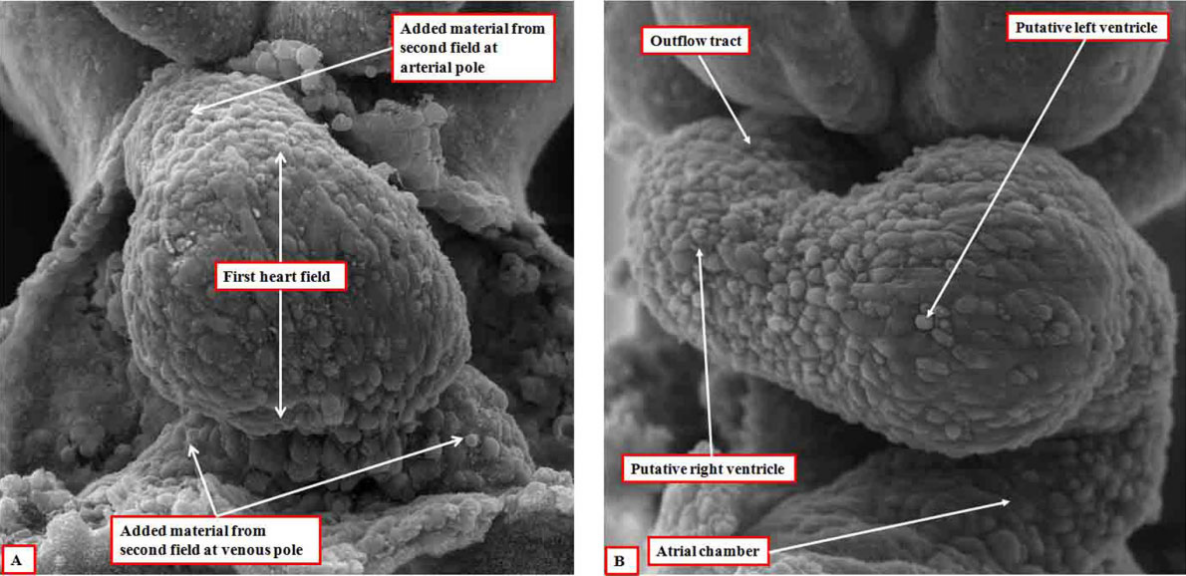
The images, prepared using dissection and scanning electron microscopy, show the developing heart tube as seen in the mouse early (panel A) and late (panel B) during the ninth day of embryonic development. This is equivalent of around five weeks of development in man.

**Figure 2.**
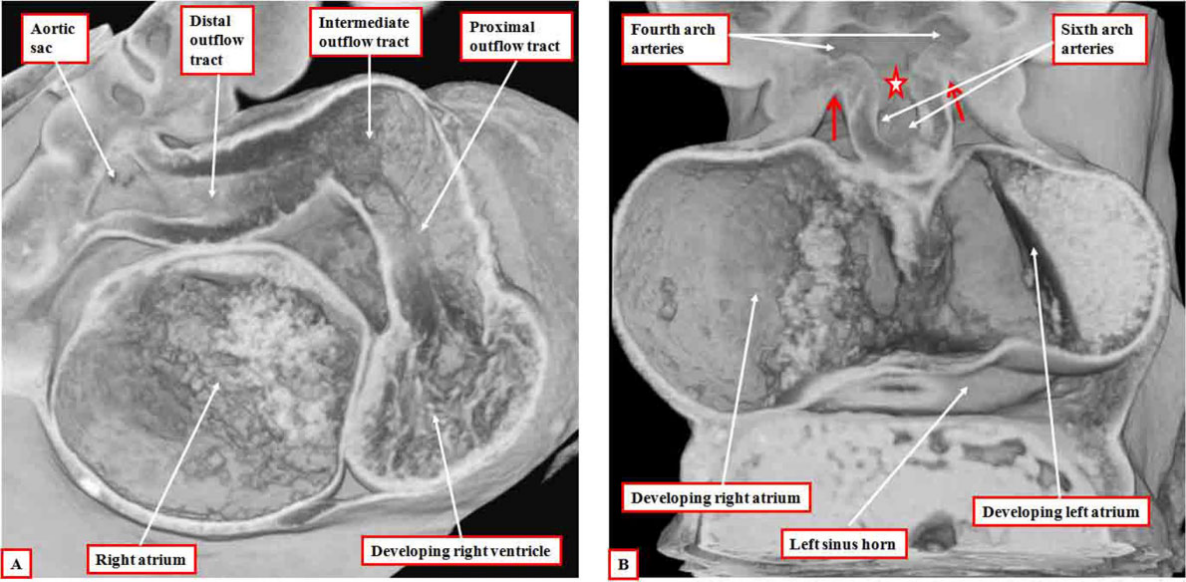
The images are from an episcopic data set prepared from a human embryo at Carnegie stage 14, representing the end of the fifth week of development. The left panel (A) shows the dog-leg bend in the outflow tract, which at this early stage is supported exclusively from the developing right ventricle. The right panel (B), cut in frontal fashion at the junction of the outflow tract with the pharyngeal mesenchyme, shows the origins of the pharyngeal arch arteries from the aortic sac. The back wall of the sac, shown by the star, is the putative aortopulmonary septum. The two single-headed arrows show the extent of the pericardial cavity.

It used to be thought that all parts of the definitive heart were represented within the initial linear heart tube. Evidence had existed for some time, nonetheless, that new cells were continuously added during development to its venous and arterial poles.^8^ These initial investigations have now been confirmed by rigorous molecular studies.^9^ The source of the newly added tissues is known as the second heart field, the initial linear tube being derived from the first heart field. The addition of the new cells from the second field to the cranial pole of the initial heart tube provides the material for formation of both the right ventricle and the outflow tract. These new cells provide not only the myocardial components of the right ventricle and the outflow tract but also the nonmyocardial intrapericardial arterial trunks, along with their valves and sinuses. When first seen, the initial heart tube is straight (Figure 1A), but with the addition of the new material, it becomes S-shaped by the process of looping (Figure 1B). As the tube elongates and loops, its lumen, throughout its length, is lined with cardiac jelly. Having looped, it retains a solitary lumen. The initial steps for the production of the eventual right- and left-sided chambers involve the process that has become known as ballooning.^10^ At the atrial level, expansions to right and left from the common atrial component of the initial tube produce the primordiums of the atrial appendages. At the level of the ventricular loop, the process involves expansions from its outer curvature. These take place in series, with the eventual apical component of the left ventricle expanding from the inlet component of the loop and that of the right ventricle expanding from the outlet part. Subsequent to looping, the outlet component of the initial heart tube extends from the cavity of the developing right ventricle to the margins of the pericardial cavity, where its initial solitary cavity becomes continuous with the aortic sac. The outlet portion then shows a significant dog-leg bend, permitting recognition of proximal, intermediate, and distal components (Figure 2A).

**Figure 3.**
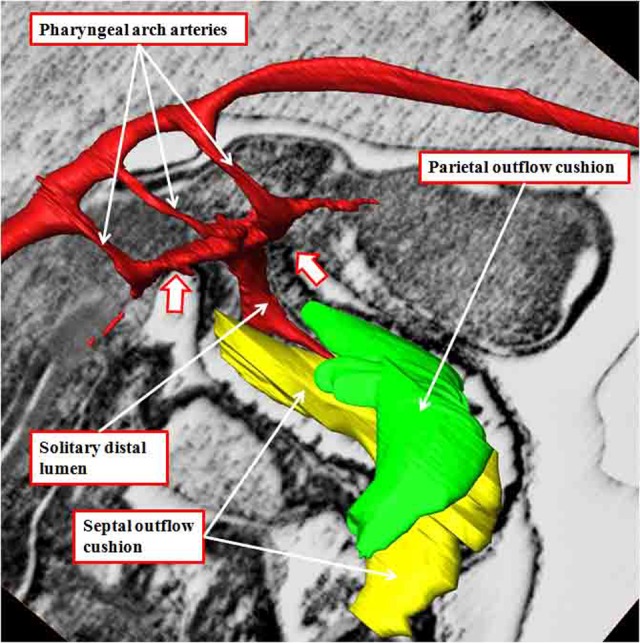
The image shows the outflow cushions reconstructed from an episcopic data set prepared from a mouse embryo killed early during the 12th day of development. The outflow cushions, shown in green and yellow, no longer reach the margins of the pericardial cavity (white arrows with red borders). The solitary lumen of the distal outflow tract becomes continuous dorsally with the cavity of the aortic sac, from which arise the pharyngeal arch arteries, at this stage bilaterally symmetrical.

At this early stage, the walls of the outflow tract are exclusively myocardial, with its lumen lined circumferentially by cardiac jelly. With the further addition of nonmyocardial tissue from the second heart field, there is regression of the distal myocardial border away from the margins of the pericardial cavity, with the contained cardiac jelly also effectively being shifted from the pericardial margins. The new nonmyocardial tissues are initially seen as cranial and caudal spurs at the pericardial border. As they extend into the pericardial cavity, the spurs rotate and form rightward and leftward tongues. These components will eventually form the parietal walls of the intrapericardial arterial trunks. As the myocardial border of the tube regresses proximally relative to the pericardial margins, the cardiac jelly lining its walls becomes converted into endocardial cushions by the process of endothelial-to-mesenchymal transformation.^11^ The outflow cushions, further populated by cells derived from the neural crest, then extend in a spiraling fashion through the intermediate and proximal parts of the outflow tract, running within the myocardial walls of these two components (Figure 3).

## Further Development of the Outflow Tract

**Figure 4.**
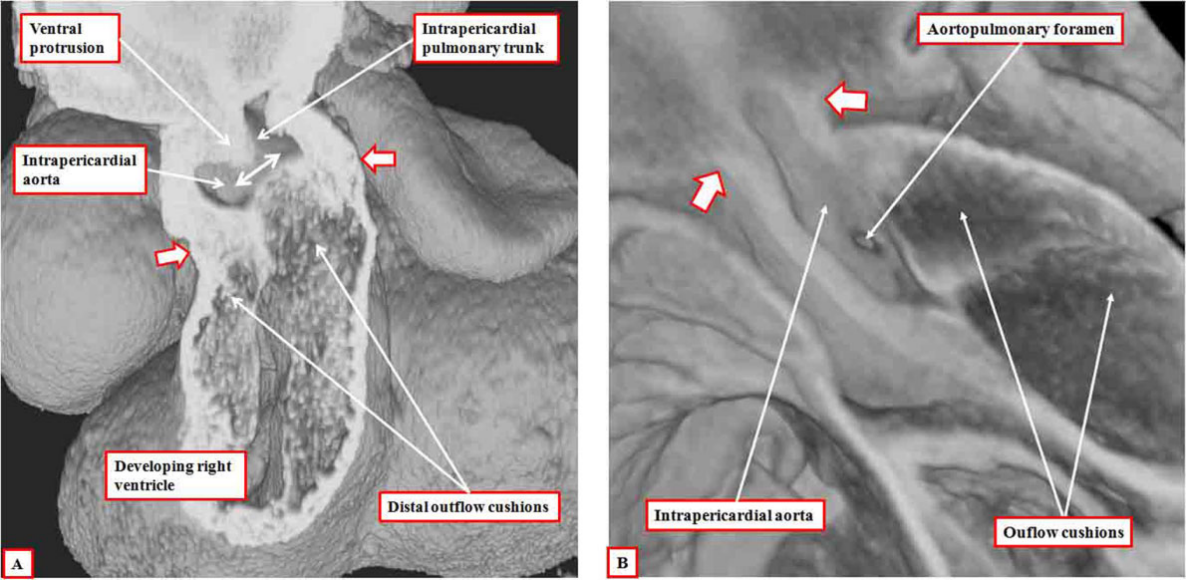
The images are both made using episcopic data sets from mice embryos killed toward the end of the 12th day of development. The left panel (A) shows a frontal section revealing the oblique nature of the ventral protrusion, which is the aortopulmonary septum. At this stage, there is an aortopulmonary foramen (double-headed white arrow) between the proximal end of the protrusion and the fused distal end of the outflow cushions. The white arrows with dark borders show the distal margin of the myocardium, which now encloses only the intermediate and proximal parts of the outflow tract. Panel B, from a different data set, shows the aortopulmonary foramen as seen from the cavity of the aorta, having cut away the parietal aortic wall. The white arrows with dark borders in this panel show the distal extent of the pericardial cavity.

**Figure 5.**
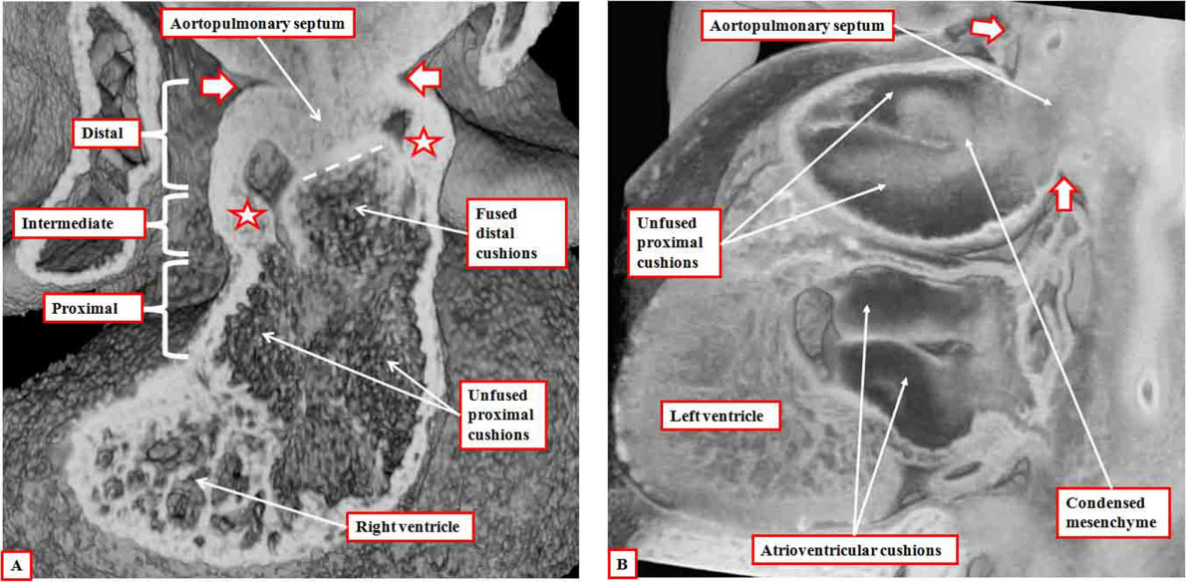
Panel A is prepared using an episcopic data set from a mouse embryo killed at the end of the 12th day of development. It shows how the aortopulmonary septum, formed by the protrusion from the dorsal wall of the aortic sac, has fused with the distal ends of the outflow cushions (dashed white line). The white arrows with dark borders show the extent of the pericardial cavity. The distal outflow tract is fully divided at this stage. The fused distal cushions have also divided the intermediate part of the outflow tract. Note the appearance of the intercalated cushions (white stars with dark borders) in this middle component. The outflow cushions, however, remain unfused in the proximal part of the outflow tract. Panel B is from an episcopic data set prepared from a human embryo at Carnegie stage 16. It shows how columns of condensed mesenchyme, derived from the cells migrating from the neural crest, occupy the fused distal and the unfused proximal parts of the outflow cushions. They are not involved with separating the intrapericardial arterial trunks, which have already been separated by the aortopulmonary septum, formed from the protrusion of the dorsal wall of the aortic sac.

The newly formed outflow cushions, which extend through the intermediate and proximal components of the outflow tract in a spiraling fashion (Figure 3), face each other throughout their length. At the initial stage, however, they have yet to fuse. Initially, therefore, the outflow tract has a common lumen. At the margins of the pericardial cavity, the lumen is continuous with the lumens of the pharyngeal arch arteries, which arise in a bilaterally symmetrical fashion from the aortic sac. The arteries extending through the fourth pharyngeal arches, which will become the systemic vessels, arise from the cranial part of the sac, with the arteries of the sixth arches, which give rise to the developing right and left pulmonary arteries, arising caudally. As the nonmyocardial tongues of tissue extend into the distal part of the outflow tract, the right-sided tongue, which will become the parietal wall of the intrapericardial aorta, is longest in cranial-to-caudal direction. The left-sided wall, destined to be the parietal wall of the pulmonary trunk, has greater length in ventrocaudal direction. Concomitant with the growth into the heart of these parietal tongues, there has been growth of the dorsal wall of the aortic sac between the origins of the fourth and sixth arch arteries. This protrusion, which is covered by cells derived from the neural crest, but with a core derived from the second heart field,^5^ grows obliquely into the distal outflow tract and forms the aortopulmonary septum (Figure 4). The cushions themselves have by now fused at their distal ends, with the proximal parts remaining unfused. Fusion of the protrusion with the distal ends of the cushions then obliterates the aortopulmonary foramen, separating the distal outflow tract into intrapericardial aortic and pulmonary channels (Figure 5A). It is only subsequent to this stage that it becomes possible to recognize columns of condensed mesenchyme, which occupy the central parts of the unfused proximal cushions (Figure 5B).

Some investigators have considered these columns, and the cushions containing them, to represent an “aortopulmonary septal complex.”^12^ In reality, the distal ends of the cushions separate the intermediate outflow tract into the arterial roots, while the subsequent fusion of the proximal cushions will divide the proximal outflow tract into the subvalvar ventricular outlets. As the fusion of the cushions proceeds progressively from distal to more proximal positions along the outflow tract, the fused tissue forms an effective septum that initially separates the aortic and pulmonary channels. Fusion is, however, rapidly succeeded by physical separation of the two outflow tracts, with discrete walls forming through the fused cushion tissue. The fusion of the embryonic aortopulmonary septum with the distal ends of the fused outflow cushions then places the right-sided intrapericardial aorta in continuity extrapericardially with the fourth arch derivatives. The same process places the extrapericardial pulmonary arteries, and the left-sided sixth arch artery, now recognizable as the arterial duct, in continuity with the intrapericardial pulmonary trunk. On the basis of presumed abnormal persistence of the different parts of the initially bilateral system of extrapericardial pharyngeal arch arteries, it is possible to explain all the various forms of vascular rings.

**Figure 6.**
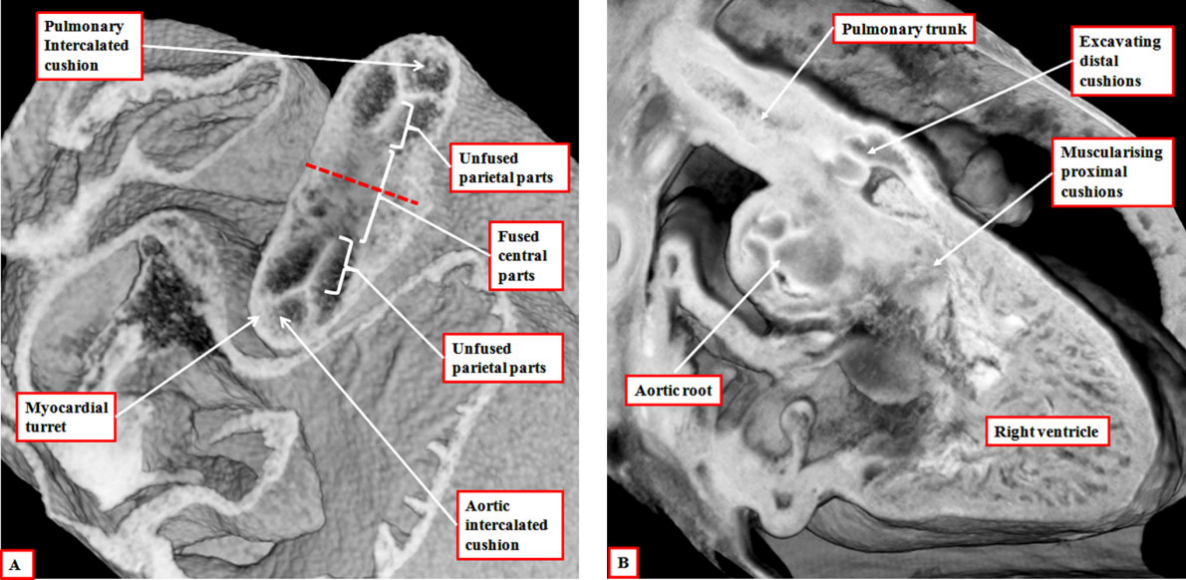
The left panel (A) is prepared using an episcopic data set from a mouse embryo killed during the 13th day of development. The short axis of the intermediate part of the outflow tract is viewed from above, showing the developing primordiums of the aortic root. Note that, at this stage, the roots remained encased in a turret of outflow tract myocardium. The right panel (B) is from an episcopic data set prepared using a human embryo at Carnegie stage 20. The three parts of the outflow tract are shown, with the distal cushions excavating to form the leaflets of the pulmonary valve, and the surface of the fused proximal cushions muscularizing to form the subpulmonary infundibulum. At this stage, the intermediate and proximal parts of the outflow tract retain their myocardial walls.

**Figure 7.**
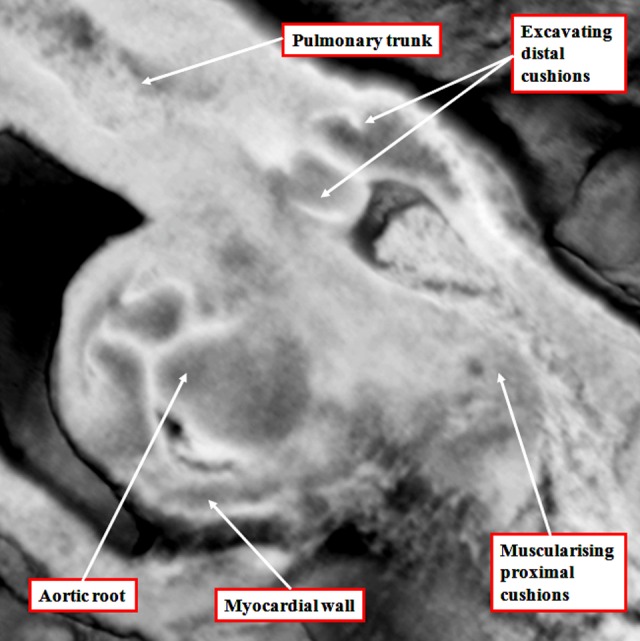
The image, a magnification of the section shown as Figure 6B, reveals the start of the excavation of the cushions that will eventually produce the leaflets of the arterial valves. At this stage, the intermediate component of the outflow tract remains encased within a myocardial turret.

Within the intermediate part of the outflow tract, still walled by myocardium, it is the appearance of the intercalated cushions (Figure 5A) that provides the primordiums for the formation of the arterial roots. Thus, within the intermediate part of the outflow tract, which gives rise to the arterial roots, fusion across the central portion of the major cushions leaves two distinct valvar primordiums clearly recognizable by their trifoliate pattern. In both cases, this is produced by the unfused edges from the parietal portions of the major cushions, interdigitated by an intercalated cushion (Figure 6A). The process of fusion itself is dependent on the presence of cells derived from the neural crest.^13^ Once fusion has taken place, however, the material derived from the neural crest becomes increasingly insignificant, eventually disappearing as the aortic and pulmonary roots separate one from the other. Separation of the roots, and also the proximal outflow tracts, occurs at right angles to the line of fusion between the central cushions. By the time the two roots have separated, the distal margins of the cushions have excavated to produce the valvar leaflets, and their semilunar hinges, these processes taking place within the supporting walls of the intermediate part of the outflow tract, which initially remain myocardial (Figures 6B and 7).

**Figure 8.**
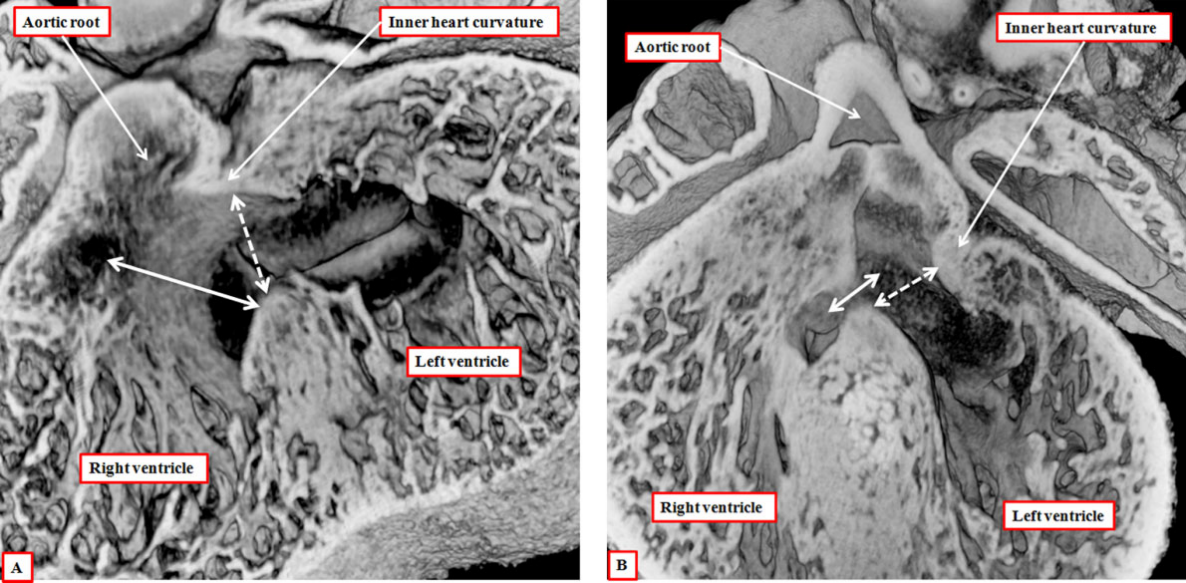
The images are from episcopic data sets prepared from developing mice killed during the 13th embryonic day (panel A) and at the beginning of the 14th day of development (panel B). They show that, as the aortic root is transferred so as to take origin from the left as opposed to the right ventricle, the plane of the initial interventricular communication (dashed double-headed white arrow) becomes the left ventricular outflow tract. This plane is roofed by the inner heart curvature. The plane from the ventricular septal crest to the fused proximal outflow cushions (double-headed solid white arrow) then becomes the interventricular communication. Note that, at this stage, the distal myocardial boundary remains confluent with the edges of the excavating distal cushions.

There is then still further migration of nonmyocardial tissues derived from the second heart field in proximal direction to produce the valvar sinuses of the arterial roots, this process in itself contributing to ongoing effective proximal regression of the distal myocardial border. Initially, therefore, the developing valvar leaflets are hinged exclusively from the myocardium. It is later in development, concomitant with the formation of the valvar sinuses, that the developing leaflets achieve their semilunar configurations, with the distal parts hinged from the newly formed arterial walls, and the levels of the most distal margins then recognizable as the developing sinotubular junctions. By the time the excavation of the leaflets is complete, the aortic root has been transferred to the left ventricle by remolding of the proximal outflow tract. This is achieved by the leftward movement of the proximal part of the outflow tract across the crest of the muscular ventricular septum, with concomitant remodeling of the embryonic interventricular communication. This communication is initially roofed by the myocardial inner heart curvature (Figure 8A). As the dorsal part of the proximal outflow tract moves toward the left ventricle, so the proximal parts of the outflow cushions, which themselves are fusing during this process, are brought into line with the crest of the muscular septum. This means that the initial embryonic interventricular communication, roofed by the inner heart curve, becomes the outflow tract for the left ventricle (Figure 8B).

**Figure 9.**
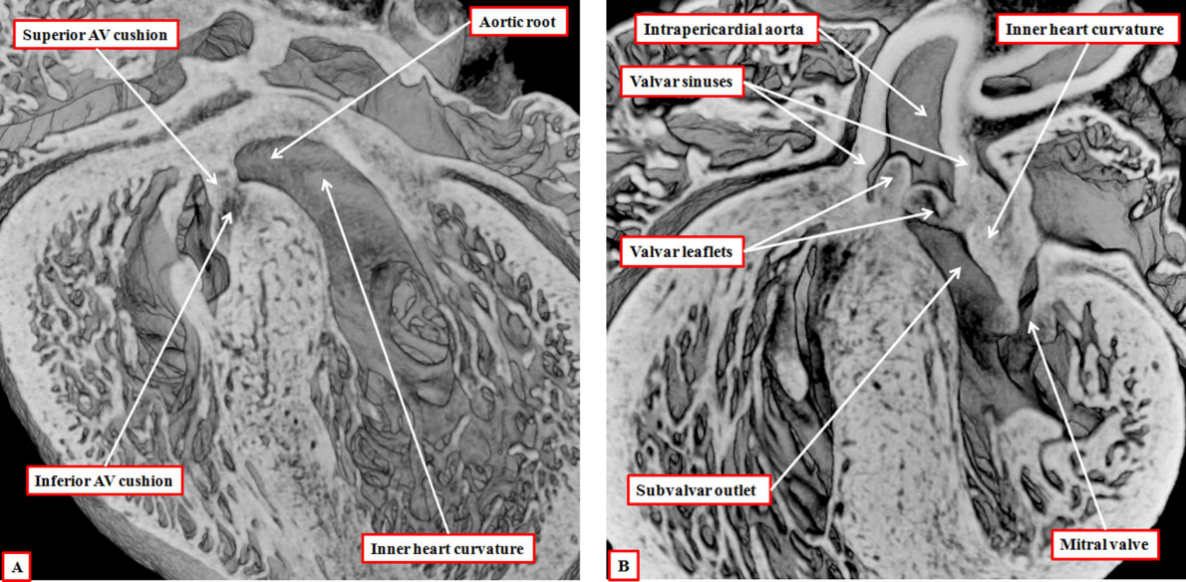
The images are from different episcopic data sets prepared from mice killed during the 15th day of embryonic development. They show, in the left panel (A), how the rightward margins of the atrioventricular (AV) cushions close the persisting interventricular communication. The right panel shows how, subsequent to closure of the foramen, the inner heart curvature remains interposed between the developing leaflets of the aortic and mitral valves. Note the developing sinuses of the aortic root and the three components of the left ventricular outflow tract.

As well as fusing during this process, the proximal outflow cushions also muscularize.^14^ The core of the cushions, packed with cells derived from the neural crest, then attenuates. The muscularized surface will eventually form the septal component of the subpulmonary infundibulum. It is the attenuation of the core that produces the extracavitary tissue plane separating the infundibulum from the newly formed aortic root. Conversion of the core of the cushions to extracavitary fibroadipose tissue means that the proximal cushions lose their initial septal configuration. The initial embryonic interventricular communication by now having remodeled to form the subaortic outflow tract, the persisting interventricular communication is closed by the formation of the membranous septum derived from the rightward tips of the atrioventricular cushions (Figure 9A). The transfer of the aorta to the left ventricle, therefore, proceeds by alignment of the muscularizing proximal outflow cushions with the apical muscular ventricular septum. When transfer is complete, and the persisting embryonic interventricular communication has been closed by the formation of the membranous septum, the aortic valvar leaflets initially remain supported in their entirety by myocardial tissues, with the inner heart curvature continuing to interpose between the leaflets of the developing aortic and mitral valves (Figure 9B). It is later during fetal development that this muscular fold becomes transformed into the region of aortic-to-mitral valvar fibrous continuity, this being one of the features of the postnatal left ventricular outflow tract.

To summarize the processes of development, from the outset, it is possible to recognize proximal, intermediate, and distal part of the outflow component of the primary heart tube. With ongoing contributions of nonmyocardial tissues from the second heart field, the distal part of the outflow tract becomes transformed into the intrapericardial trunks. The cavities of the trunks are initially separated by an embryonic aortopulmonary septum, but this disappears as the intrapericardial aorta and pulmonary trunk develop their own discrete walls. The intermediate part of the outflow tract, subsequent to the formation within it of the intercalated cushions, becomes transformed into the arterial roots. The distal cushions, along with the intercalated cushions, excavate to form the semilunar valvar leaflets, whereas the valvar sinuses are formed from ongoing contributions of nonmyocardial tissues from the second heart field. As the roots separate one from the other, so the intermediate cushions lose their initial septal function. The proximal cushions fuse both with each other and the crest of the muscular ventricular septum. It is the muscularization of their surface that produces the dorsal component of the right ventricular infundibulum (Figure 6B). Only after the aortic component of the proximal outflow tract has been transferred to the left ventricle, can there be closure of the remodeled embryonic interventricular communication (Figure 9A). And only after this has taken place, does the left ventricular component of the inner heart curvature undergo transformation to produce the area of aortic-to-mitral fibrous continuity that forms the roof of the definitive left ventricle.

## Morphology of the Postnatal Outflow Tracts

**Figure 10.**
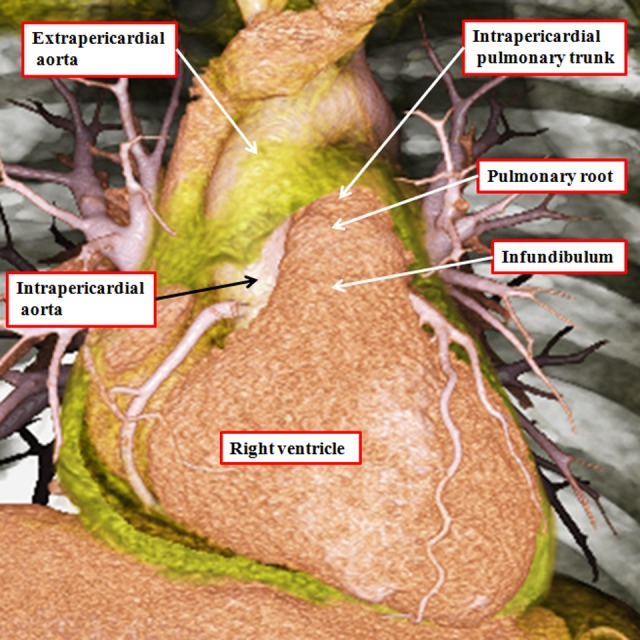
The image is prepared from a data set obtained using multidetector computed tomography in a patient undergoing investigation for coronary arterial disease. The pericardial reflections divide the ascending aorta into intrapericardial and extrapericardial components. The pulmonary trunk branches at the margins of the pericardial cavity into the right and left pulmonary arteries.

**Figure 11.**
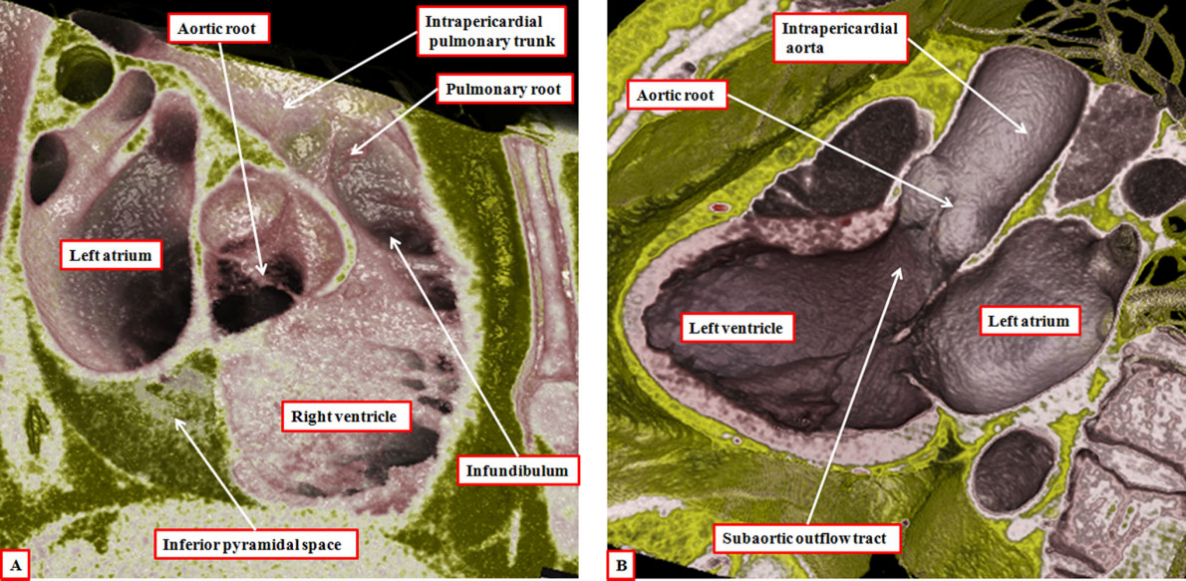
The virtual dissections are made from a data set obtained using multidetector computed tomography in a patient undergoing investigation for coronary arterial disease. They show how each outflow tract is formed in a tripartite fashion, with the components represented by the intrapericardial arterial trunk, the arterial root, and the subvalvar outflow tract, respectively. Note that, in the right ventricle (panel A), the subvalvar component is a completely muscular infundibular sleeve, whereas in the left ventricle (panel B), the posterior wall of the subvalvar area is formed by fibrous continuity between the leaflets of the aortic and mitral valves.

**Figure 12.**
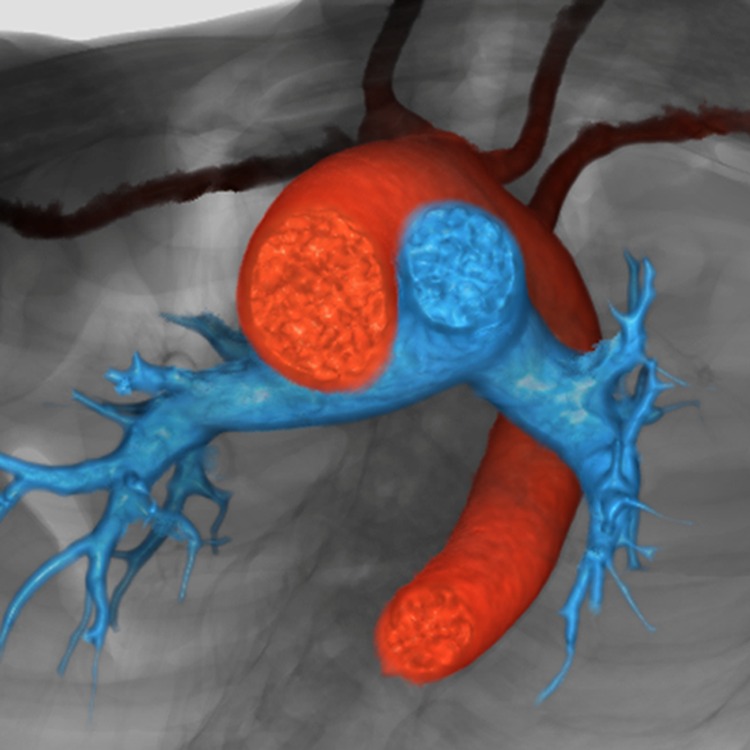
The reconstruction, seen in short-axis projection from the cardiac apex, shows the spiraling nature of the intrapericardial arterial trunks. The aorta is shown in red, and the pulmonary trunk in blue.

Just as with the investigation of the developing heart, the emergence of new diagnostic techniques has improved our ability to demonstrate the nuances of living cardiac anatomy. To achieve this, we have taken advantage of the preparation of three-dimensional data sets acquired during the investigation of coronary arterial disease to perform virtual dissections of normal human hearts.^15^ These images not only serve to validate our earlier descriptions of the morphology of the intrapericardial outflow tracts,^16-18^ but at the same time they confirm the tripartite arrangement of their constituent parts. They also show well the locations of the pericardial reflections that divide the arterial trunks into their intrapericardial and extrapericardial parts (Figure 10). Further virtual dissection of the data sets then shows well how both of the ventricular outflow tracts possess three parts. These are the intrapericardial component of the arterial trunk, the arterial root, and the subvalvar ventricular outflow tract. In the morphologically right ventricle, the subvalvar component is a complete muscular sleeve, which lifts the root away from the cardiac base, whereas in the left ventricle, the posterior wall of the outflow tract is fibrous, being made up of fibrous continuity between the leaflets of the arterial and atrioventricular valves (Figure 11). In the normal heart, the intrapericardial arterial trunks spiral as they extend from the arterial roots to the margins of the pericardial cavity (Figure 12). Although the developing intrapericardial trunks had initially been separated by the aortopulmonary septum (Figure 4A), we know that the central core of the embryonic septum is attenuated by term, so that, in postnatal life, the trunks have their own discrete walls, with no septal component between them.

**Figure 13.**
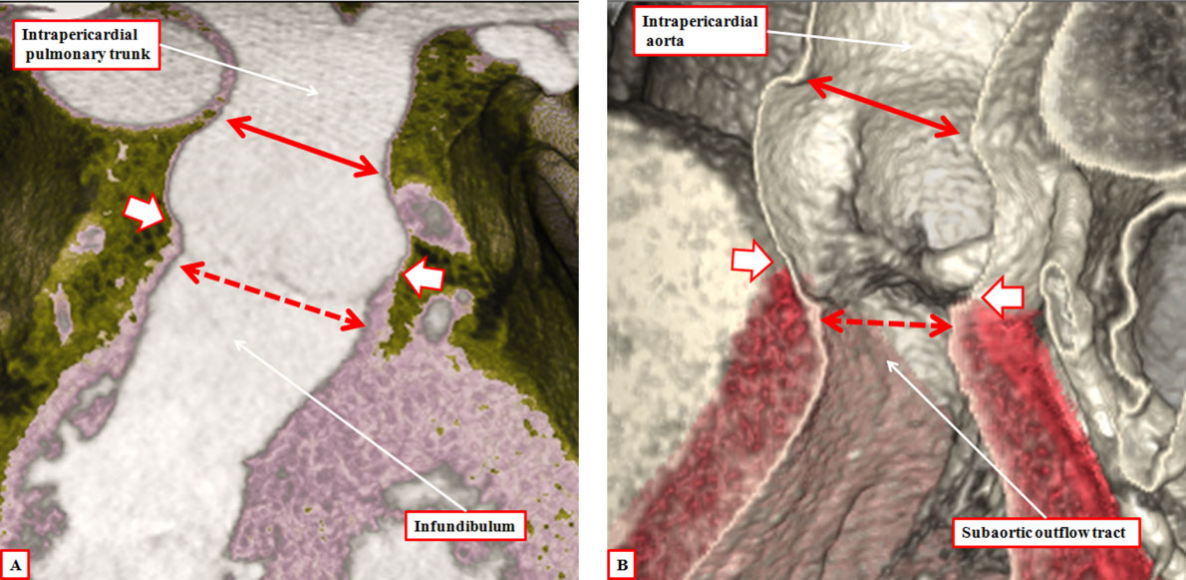
The virtual dissections show how the pulmonary (panel A) and aortic (panel B) roots are limited distally by the sinotubular junctions (solid double-headed arrow) but proximally by a virtual plane made by joining together the basal attachments of the semilunar leaflets (double-headed dashed arrow). Note that the virtual basal plane is proximal to the anatomic ventriculoarterial junctions (white arrows with dark borders).

**Figure 14.**
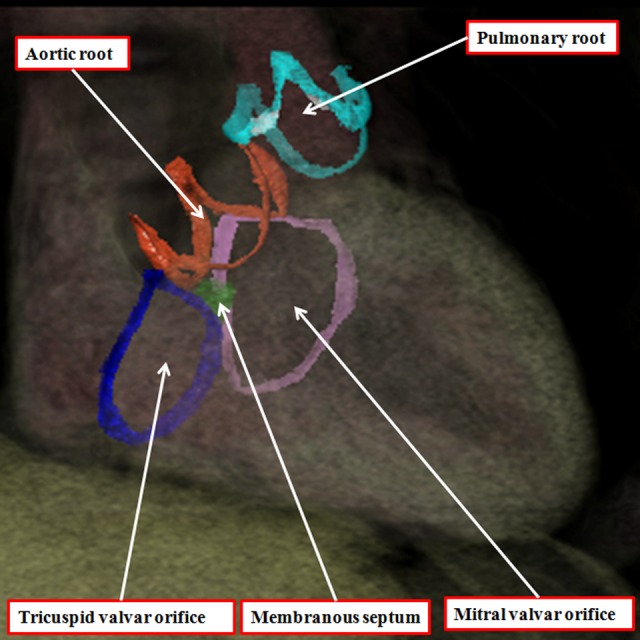
The three-dimensional data set made available by means of multidetector computed tomography from a patient undergoing investigation of coronary arterial disease has been segmented and reconstructed to show the hinges of the leaflets of the arterial and atrioventricular valves. The leaflets of the atrioventricular valves are hinged in an annular fashion, whereas the hinges of the arterial valves, when reconstructed, take the form of three-pointed coronets.

The arterial roots occupy the middle parts of the outflow tracts and are limited distally by the sinotubular junctions, there being the areas where the semilunar hinges of the arterial valvar leaflets come together at the periphery of the valvar orifices (Figure 13). There is no discrete anatomical structure that marks the proximal boundaries of the arterial roots. Instead these are represented by virtual planes constructed by joining together the proximal attachments of the hinges of the valvar leaflets. These virtual basal planes, furthermore, are proximal to the anatomic ventriculoarterial junctions. The latter boundaries, true anatomic borders, are formed at the points where the walls of the valvar sinuses are supported by the ventricular walls. In the right ventricle, the entirety of the walls of the arterial valvar sinuses is supported by infundibular musculature.^17^ In the aortic root, in contrast, because the fibrous continuity found posteriorly between the leaflets of the aortic and mitral valves, only the two aortic valvar sinuses giving rise to the coronary arteries have myocardium incorporated into their bases (Figure 13B). The entrances to the arterial roots, therefore, have no discrete anatomic boundaries. These are, nonetheless, the dimensions that are usually measured by echocardiographers as representing the valvar “annulus.” The reconstructions made possible by the availability of the three-dimensional data sets show that the hinges of the leaflets of the arterial valves, unlike those of the atrioventricular valves, are arranged in the form of three-pointed coronets rather than as little rings (Figure 14).

These features produce the problems that continue with regard to the definition of the arterial valvar annuluses. As stated above, echocardiographers define the virtual basal plane as the annulus, even though it is not marked by any anatomic entity. In contrast, many, but not all, surgeons consider the semilunar hinge lines to represent the annulus. This is one of the disagreements contributing to the “tower of Babel” currently existing with regard to the naming of the arterial roots.^19^ One of us had already expressed his own opinion that the virtual plane is best considered as the annulus,^20^ not least since this is the measurement most frequently provided to surgeons when assessing valvar morphology. The virtual plane, furthermore, is also annular, unlike the coronet-like arrangements of the valvar hinges.

**Figure 15.**
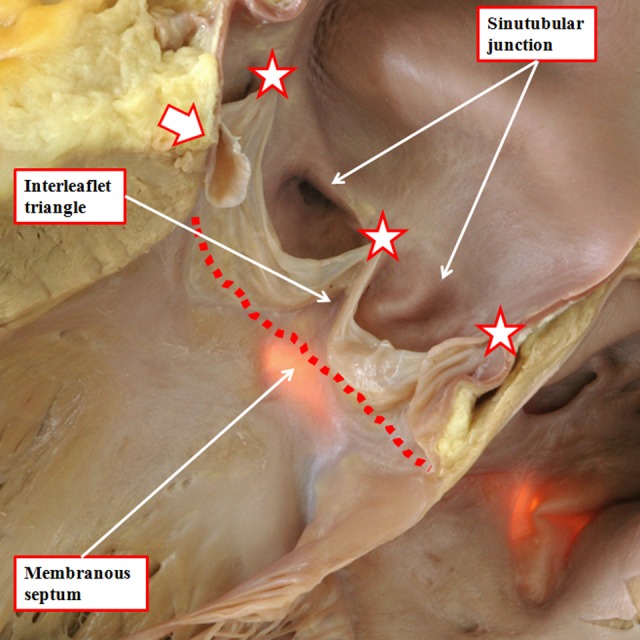
The outflow tract of the normal heart has been opened through an incision across the left coronary sinus of the aortic root. The membranous septum has been transilluminated from the right side. This shows how the triangular space between the right coronary and the nonadjacent sinuses of the root is filled by a fibrous wall. Similar triangles with fibrous walls are to be found beneath each of the valvar commissures, these being the points at which the leaflets join together at the sinotubular junction (white stars with dark borders). The relationships of these triangles are shown in Figure 16. Note how the virtual basal plane is created by joining together the basal attachments of the valvar leaflets (dotted line).

**Figure 16.**
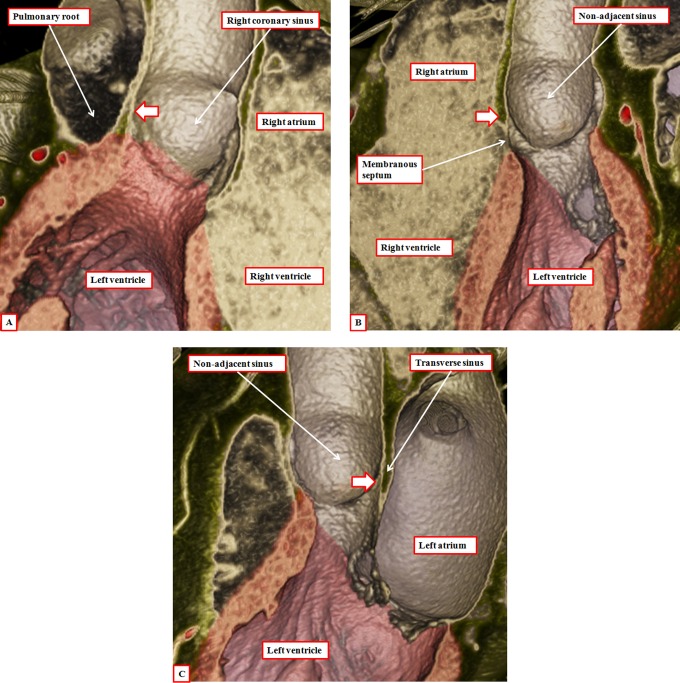
Virtual dissection of the aortic root shows how the tips of each of the interleaflet triangles (white arrows with dark borders) point outside the cardiac cavities. Panel A shows a cut through the triangle between the right and left coronary aortic sinuses and is viewed from behind. Panel B shows a cut across the triangle between the right coronary and the nonadjacent aortic sinuses and is shown from the front. Note that the membranous septum forms the base of this triangle (see also Figure 15). Panel C, showing a cut through the triangle between the left coronary and the nonadjacent aortic sinuses, is viewed from the left side.

Another problem contributing to the tower of Babel is the variable use of the word “cusp” when describing the components of the root. Some use the word to describe the leaflets, whereas others use it to account for the valvar sinuses. Our own preference is to avoid its use altogether, since when used in the vernacular sense, it accounts for a point or the crossing of two curves. It is by distinguishing between leaflets and sinuses, and avoiding the use of cusp, that we are able to provide a more accurate account of the morphology of the arterial roots. Assessment in this fashion also serves to emphasize the significance of the fibrous interleaflet triangles,^21^ which fill the spaces on the ventricular aspect of the sinuses. It is the sinuses themselves, of course, that support the distal parts of the valvar leaflets in semilunar fashion (Figure 15). During development, as we have shown, the entirety of the developing arterial roots is enclosed with the myocardial wall of the intermediate part of the outflow tract (Figure 5A). As we have also shown, the myocardial border regresses toward the cardiac base concomitant with the formation of the nonmyocardial valvar sinuses (Figure 9B). It is because of the myocardial regression that the apexes of the interleaflet triangles, in postnatal life, are able to point to extracardiac areas. These are the tissue plane between the aortic root and the infundibulum and the transverse pericardial sinus (Figure 16). Recognition of the relationships of these triangles is achieving increasing importance with the realization that many patients with acquired disease of the aortic valve can undergo reconstructive procedures as opposed to valvar replacement.^22^ The triangles are poorly formed and hypoplastic in the setting of congenital bicuspid and unicuspid valves (see below).

**Figure 17.**
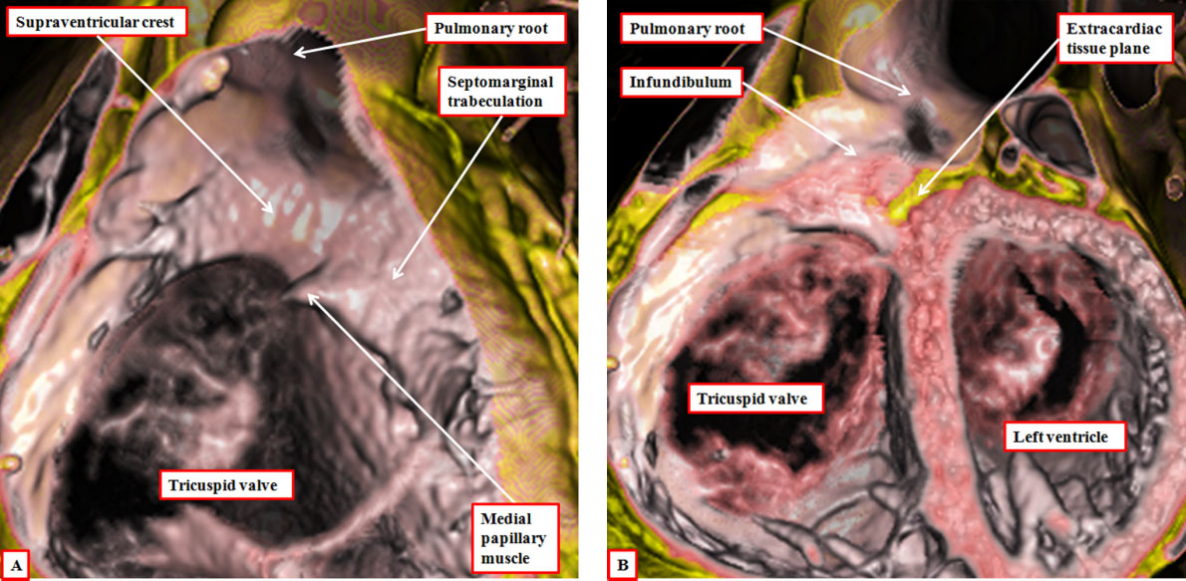
The images show virtual dissections revealing the structure of the right ventricular infundibulum. The left panel (A) is made by cutting away the parietal wall of the right ventricle, showing how the posterior wall of the infundibulum is formed by the supraventricular crest. The larger part of this wall is no more than the inner heart curvature. The component adjacent to the aortic root, however, is the free-standing infundibular sleeve, the presence of which makes it possible to remove the root for use as an autograft in the Ross procedure. The right panel (B) shows this sleeve, revealing the tissue place that separates it from the aortic root.

The third component of the outflow tracts is the outlet parts of the ventricles. As we have emphasized, these differ in the two ventricles, since the right ventricular outlet is a completely muscular infundibular sleeve. The anterior part of the sleeve is the parietal wall of the right ventricle, with the posterior part formed by the supraventricular crest (Figure 17). In the past, we considered the entirety of the posterior part of the infundibulum to be formed by the muscular outlet septum.^23^ We know now that this is not the case.^6^ The core of the proximal outflow cushions, which initially formed an embryonic outlet septum, attenuates subsequent to muscularization of their surface to produce the posterior wall of the infundibulum (Figure 6B). At the site of the attenuating tissues, we eventually find the extracavitary fibroadipose tissue that interposes postnatally between the newly formed infundibulum and the aortic root (Figure 17B).

## Correlations With Outflow Tract Malformations

We have already discussed how interpretation of inappropriate remodeling and attenuation of the initially bilaterally symmetrical arrangement of the arteries extending through the pharyngeal arches is able to provide rational explanations for the various lesions known as vascular rings and slings. These are malformations of the extrapericardial arterial trunks, including the enigmatic fifth arch artery.^24^ It is the remodeling of the extrapericardial arterial trunks and their branches that provides alternative explanations for several of the lesions currently interpreted on the basis of retention of the fifth arch arteries. One of the problems with the conventional interpretations is that neither the alleged fifth pharyngeal arch nor its supposed artery is to be found in developing murine embryos.^25^ A remnant of the artery, nonetheless, has been found in one developing human embryo,^24^ so the conventional interpretations can remain valid, although there are better explanations for some of the lesions interpreted in this fashion.^25^ All of the lesions interpreted on the basis of persistence of the fifth arch artery, nonetheless, along with lesions involving persistent patency of the arterial duct, are extrapericardial malformations.

**Figure 18.**
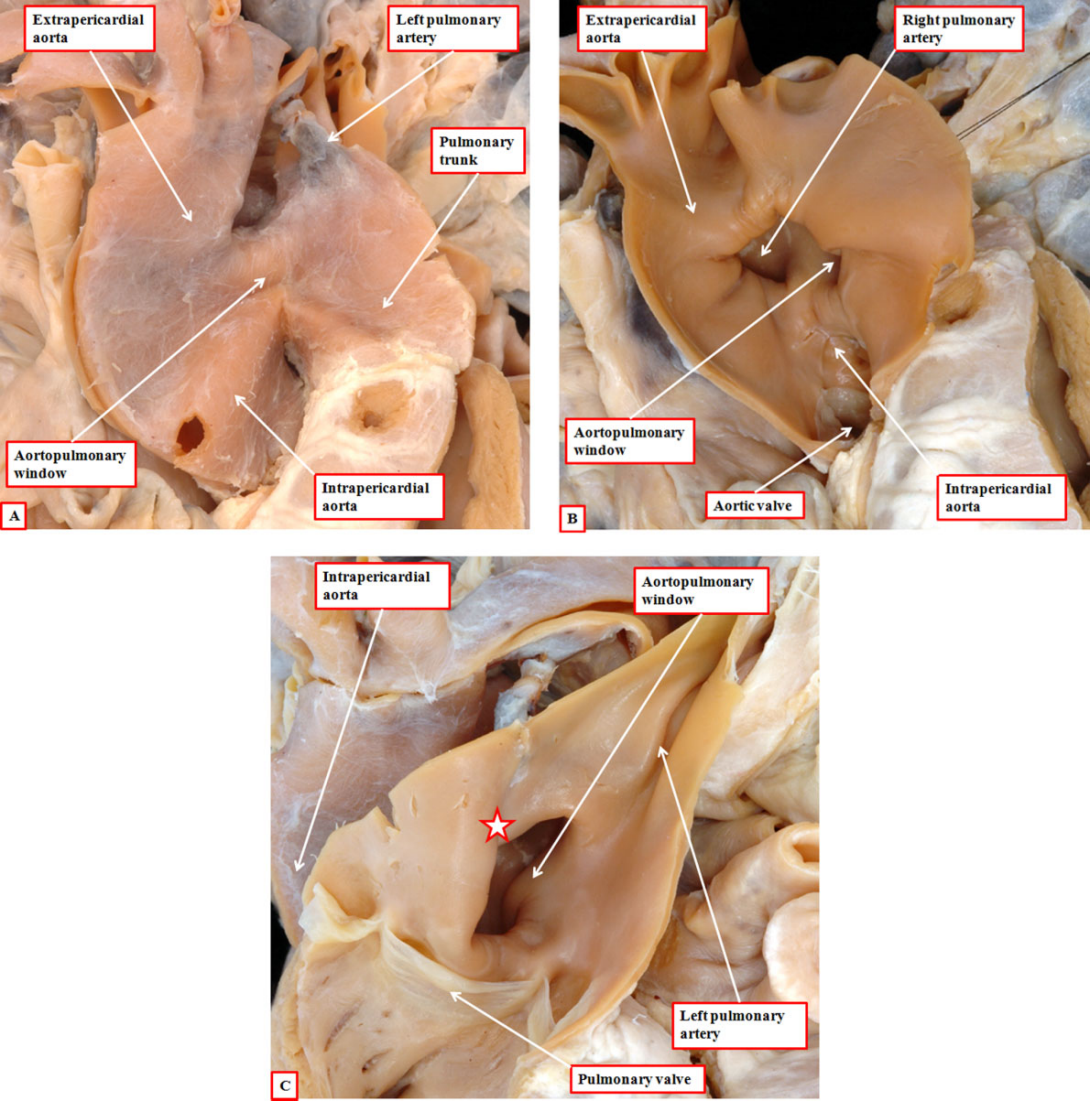
The images show an aortopulmonary window (panel A), with origin of the right pulmonary artery on the aortic side of the window (panel B). This is well explained by inadequate growth of the aortopulmonary septum from the dorsal wall of the aortic sac (see Figure 4A), leaving a fold between the walls of the aorta and the pulmonary trunk at the margins of the pericardial cavity (star in panel C). Note the separate formation of the aortic and pulmonary roots, implying normal septation and separation of the intermediate and proximal components of the developing outflow tract.

Lesions such as aortopulmonary window, or anomalous origin of the right pulmonary artery from the aorta, are intrapericardial. They are best explained on the basis of failure to close the embryonic aortopulmonary foramen, this in turn reflecting inadequate growth of the aortopulmonary septum from the dorsal wall of the aortic sac. Failure of the latter process explains well why patients with aortopulmonary window are frequently found with the right pulmonary artery arising from the aortic side of the window (Figure 18). Eccentric growth, but subsequent fusion, of the aortopulmonary septum with the distal ends of the outflow cushions provides a plausible explanation for origin of the right pulmonary artery from the ascending aorta. The separate origins of the aortic and pulmonary roots in this lesion point to the inadequacy of using the term “hemitruncus” for its description.

**Figure 19.**
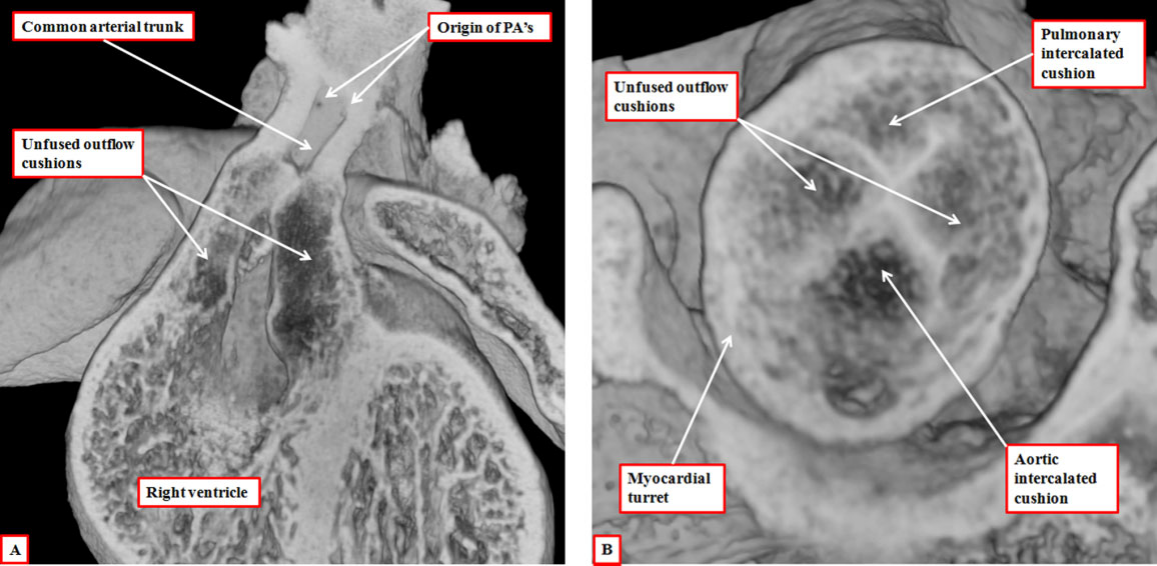
The images are from embryonic mice in which the gene for Tbx1 has been knocked out. All these mice develop with common arterial trunks. The images are from embryos killed on the 13th day of development. The left panel (A) shows the common trunk, feeding the systemic and pulmonary arteries, with no formation of the aortopulmonary septum. It also shows failure of fusion of the outflow cushions. The right panel, from a different embryo at the same stage of development, shows a cross section through the intermediate part of the outflow tract. Both the intercalated cushions are seen, together with the distal ends of the unfused major outflow cushions. This template provides the primordiums for the formation of a common truncal valve with four leaflets. Note that the cushions are contained within the turret of myocardium that surrounds the intermediate part of the outflow tract.

The essence of hearts having a common arterial trunk is the commonality of the ventriculoarterial junction. This finding points to another of the problems of using truncus and conus for describing the components of the developing outflow tract. It is the ventriculoarterial junctions that are common in this setting of common arterial trunk rather than the arterial trunks. When accounting for development in tripartite fashion, there are no such problems, since we know that the abnormal mechanism is failure of fusion of the major outflow cushions (Figure 19). It had previously been suggested that the mechanism for formation of common arterial trunk, or “truncus arteriosus communis,” was failure of formation of the subpulmonary outflow tract.^26^ Van Mierop and colleagues, having studied a Keeshond dog exhibiting the lesion, showed that the morphogenetic culprit was failure of fusion of the major cushions within the developing outflow tract.^27^ Our studies using a colony of mice with knockout of the *Tbx1* gene have confirmed their observations.^6^ The presence of both intercalated cushions in hearts destined to produce common arterial valves with four leaflets and sinuses adds further weight to the fact that the lesion reflects failure of septation of the outflow tract rather than inadequate formation of its pulmonary component. We have, nonetheless, also observed developing embryos with gross hypoplasia of one of the intercalated cushions, this feature providing the template for the formation of the trifoliate common truncal valve.^6^

**Figure 20.**
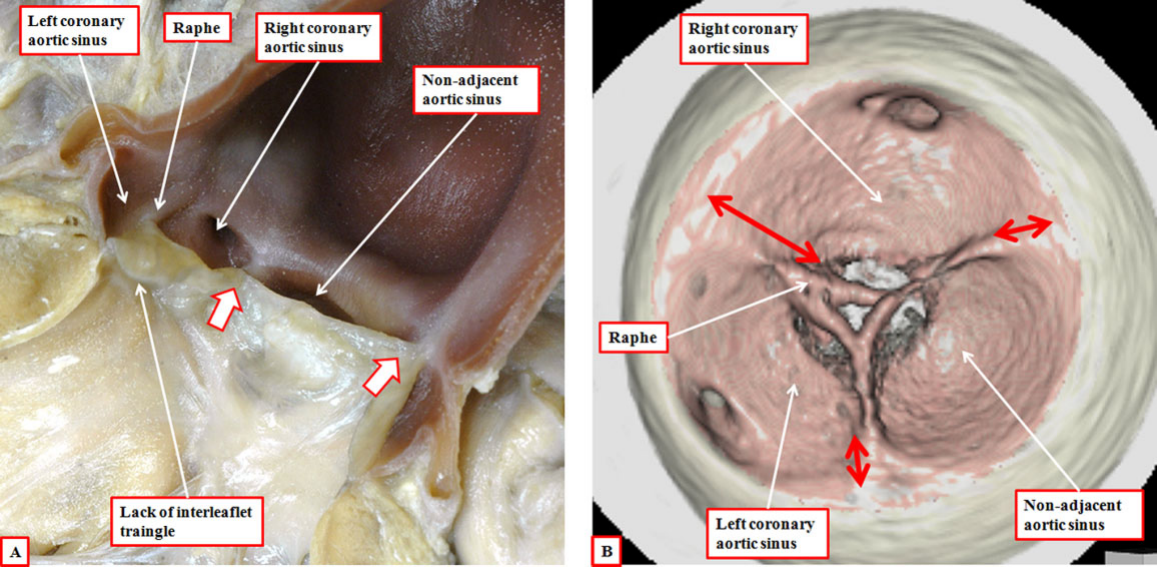
In panel A, we show the features of the bicuspid aortic valve with the conjoined leaflet derived from the leaflets that usually guard the valvar sinuses giving rise to the coronary arteries. There is formation only of the interleaflet triangles between the nonadjacent leaflet and the conjoined leaflet (white arrows with dark borders), with a raphe formed at the anticipated site of the interleaflet triangle between the two coronary aortic sinuses. Panel B shows reconstruction of the hinges of the valvar leaflets in an adult with a bicuspid aortic valve and conjunction of the leaflets guarding the coronary aortic sinuses. The anticipated point of the coronet at the site of the fused leaflets does not extend to the sinotubular junction (long double-headed arrow), in contrast to the zones of apposition with the other leaflet, which extend to the sinotubular junction (short double-headed arrows).

Failure of fusion of the outflow cushions, therefore, is responsible for producing the common arterial trunk, this lesion being well accepted as a conotruncal anomaly. Excessive fusion of the ends of the major outflow cushions is then the likely causative factor in producing the aortic valve with two leaflets, the conjoined leaflet being formed from the leaflets that normally guard the aortic valvar sinuses giving rise to the coronary arteries (Figure 20A).^28^ Fusion between one of the major cushions and the aortic intercalated cushion then provides an explanation for the bicuspid valve with the conjoined leaflet derived from the right coronary and nonadjacent aortic valvar leaflets.^29^ Our reconstructions of the valvar hinges in adult patients with bicuspid aortic valves confirm the lack of formation of the interleaflet triangle, with the hinges at the site of fusion of the developing leaflets failing to rise to the sinotubular junction. Since the morphogenesis of the bicuspid arterial valves is excessive fusion of the developing outflow cushions, we see no reason why these lesions, and the others involving abnormal development of the arterial roots, should not be included in the category of conotruncal anomalies.

**Figure 21.**
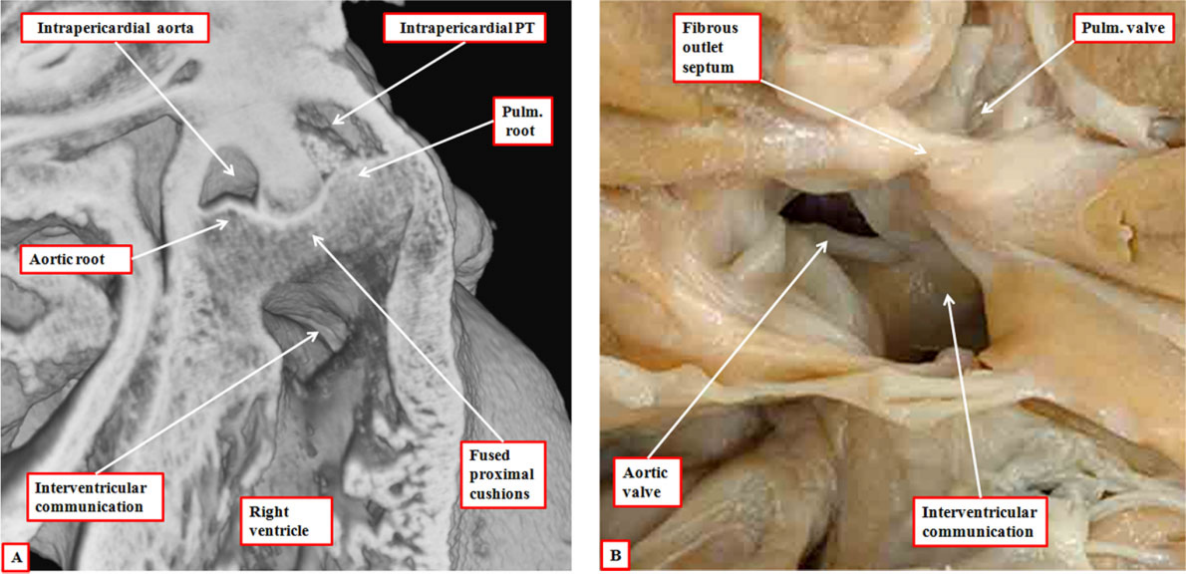
The left panel (A) is taken from an episcopic data set prepared from a developing mouse embryo at the 15th day of development with knockout of the Furin enzyme. The aortopulmonary septum has fused with the distal cushions to produce separate intrapericardial arterial trunks. The outflow cushions themselves are also fused, but with minimal development of the proximal components, which have failed to muscularize. As a result, the arterial trunks remain supported by the right ventricle, the interventricular communication being doubly committed. This arrangement is seen postnatally in the heart with double outlet right ventricle shown in the right panel (B). It has a fibrous rather than a muscular outlet septum. The interventricular communication, opening to the right ventricle between the limbs of the septomarginal trabeculation, is doubly committed.

As well as lesions involving the distal and intermediate components of the developing outflow tracts, many congenital malformations are due to maldevelopment of the proximal outflow tract. Since, when first formed, the developing outflow tract is supported exclusively by the developing right ventricle, it is hardly surprising that double outlet right ventricle is one of these lesions. Double outlet right ventricle, however, is no more than an abnormal ventriculoarterial connection. Multiple phenotypes are to be found in this setting, most frequently categorized according to the location of the interventricular communication relative to the arterial trunks. This channel between the ventricles is most usually described as the “ventricular septal defect.” When both arterial trunks arise postnatally from the right ventricle, the channel is roofed by the inner heart curvature. In this setting, the hole is then the outflow tract from the right ventricle, rather than representing the area to be closed so as to produce potential biventricular circulations. The channel that would, most appropriately, be termed the ventricular septal defect would be roofed by the outlet septum, or its fibrous remnant. The attachments of this outlet septum, or its remnant, within the right ventricle, furthermore, determine the relationships between the interventricular communication and the arterial roots. When attached to the anterior margin of the defect, it is the aortic root that is closest to the left ventricle, whereas when attached to the posterior margin, the defect is placed in subpulmonary location. If, however, the developing outlet septum remains hypoplastic, with minimal formation of the proximal outflow cushions, but with fusion of the distal cushions, then the defect can be doubly committed. This abnormal mechanism of development was seen in our colony of mice in which we perturbed the Furin enzyme (Figure 21A). It is comparable with the variant of double outlet right ventricle with a fibrous rather than a muscular outlet septum (Figure 21B). In terms of morphogenesis, the lesion is more akin to common arterial trunk, but with a common ventriculoarterial junction divided into aortic and pulmonary components, as opposed to the other variants of double outlet right ventricle. The feature of the latter lesions is the separate nature of the subpulmonary and subaortic ventricular outflow tracts. The variant with the subpulmonary defect is itself related developmentally to hearts with concordant atrioventricular and discordant ventriculoarterial connections, being part of the Taussig-Bing spectrum.^30^ The variants with subaortic defects, in contrast, are parts of the spectrums including the Eisenmenger defect and tetralogy of Fallot.^31^

When discussing double outlet right ventricle, it is also pertinent to consider the role of the inner heart curvature. For many years, it was considered that persistence of the inner heart curvature, in other words presence of “bilateral conuses,” was the essence of double outlet right ventricle. As we have shown, however, the inner heart curvature remains muscular even after the aorta has been transferred to the left ventricle in the setting of normal development. There is no justification, therefore, for considering persistence of the inner curvature as the phenotypic feature of double outlet right ventricle. Persistence of the inner curvature, also known as the “bulboventricular flange,” has also been correlated with the formation of the anterolateral muscle bundle of the left ventricle.^32^ Our findings suggest that the inner curve, prior to its conversion to fibrous tissue, forms no more than the roof of the left ventricle. It seems more likely that the anterolateral muscle bundle, when present, is derived by compaction of the trabecular layer of the ventricular walls. Like many of our suggestions, nonetheless, we accept that such a presumption is speculative.

## Conclusions

Problems continue to exist when seeking logically to correlate the morphogenesis of congenitally malformed hearts with concepts of normal cardiac development and also with categorizations of the so-called conotruncal malformations. In no small part, these relate to the ongoing practice of analyzing the outflow tract in terms of the truncus and the conus. The intrapericardial outflow tracts as seen in the postnatal heart unequivocally possess three components, namely, the intrapericardial arterial trunks, the arterial roots, and the subvalvar ventricular outflow tracts. As we have shown in our review, the problems outlined above are dissipated when development of the outflow tract is similarly analyzed in a tripartite fashion. Thus, it is the distal part of the outflow tract that separates to form the intrapericardial arterial trunks. The arterial roots are formed within the intermediate part of the outflow tract, initially with myocardial walls, which become arterial concomitant with ongoing addition of nonmyocardial tissues from the second heart field. It is the proximal outflow tract that produces the subvalvar outflow tracts. This component is initially supported exclusively by the developing right ventricle. Normal development requires transfer of its posterior part to the left ventricle, thus forming the subaortic outflow tract, with muscularization of the surface of the proximal outflow cushions then producing the posterior part of the newly formed subpulmonary right ventricular infundibulum. Analysis of congenitally malformed hearts is then easily achieved on the basis of the tripartite template. Such analysis reveals the need to reconsider those lesions categorized as representing conotruncal anomalies. It is paradoxical in this regard that common arterial trunk, formed because of failure of fusion of the outflow cushions, is currently included within the conotruncal category, yet arterial valves with two leaflets, representing excessive fusion of the cushions, are currently excluded.
